# A rough set approach for determining weights of decision makers in group decision making

**DOI:** 10.1371/journal.pone.0172679

**Published:** 2017-02-24

**Authors:** Qiang Yang, Ping-an Du, Yong Wang, Bin Liang

**Affiliations:** 1 School of Mechatronics Engineering, University of Electronic Science and Technology of China, Chengdu, China; 2 Southwest China Institute of Electronic Technology, Chengdu, China; Southwest University, CHINA

## Abstract

This study aims to present a novel approach for determining the weights of decision makers (DMs) based on rough group decision in multiple attribute group decision-making (MAGDM) problems. First, we construct a rough group decision matrix from all DMs’ decision matrixes on the basis of rough set theory. After that, we derive a positive ideal solution (PIS) founded on the average matrix of rough group decision, and negative ideal solutions (NISs) founded on the lower and upper limit matrixes of rough group decision. Then, we obtain the weight of each group member and priority order of alternatives by using relative closeness method, which depends on the distances from each individual group member’ decision to the PIS and NISs. Through comparisons with existing methods and an on-line business manager selection example, the proposed method show that it can provide more insights into the subjectivity and vagueness of DMs’ evaluations and selections.

## Introduction

The aim of a multiple attribute decision-making (MADM) problem is to obtain alternatives’ rankings or an optimal alternative selection by the decision information from each DM with respect to amount of criterias. Nowadays, MADM problems have been involved in various aspects of politics, economies, science, technology, culture, education and other fields [1–8].

However, along with the constantly expansion of criterias, it is nearly impossible for a single decision maker to make an appropriate judgment independently for a project [9–14]. Therefore, many companies and groups prefer to make a final decision through a panel of experts [15–20]. Each expert has his/her preference to each attribute based on his/her knowledge level and cognitive capability. As the preference information of each expert is always different in group decision-making problems, current research focus on the aggregation of decision information and priority order of group members [21].

French [22] proposed three major postulates and a variety of theorems to deal with the effects of group members’ opinions. Theil [23] proposed an approach to define the weights of the linear combination of individual preference functions in committee decision problem. Bodily [24] developed a delegation process to setting the members’ weights, which is obtained using the theory of Markov chains. Mirkin and Fishburn [25] make use of eigenvectors method to gain weights information of group members. Martel and Ben Khelifa [26] use individual outranking criterias to determine the group members’ weights. Ramanathan [27] developed an AHP method to obtain group members’ weights, and aggregated group decisions. Fu and Yang [28] used a group consensus to address multiple attributive group decision problems, which is from evidential reasoning approach. Xu and Wu [29] proposed a discrete model to support the group consensus reaching process, in which the weights of experts is pre-defined. Zhou et al. [30] proposed the generalized logarithm chi-square method to aggregate group members’ information. Zhang [31] presented several generalized Atanassov’s intuitionistic fuzzy power geometric operators to aggregate input arguments. Yue [32] presented an extended TOPSIS method for ranking the order of decision makers and the order of alternatives. Efe [33] proposed an integration of fuzzy AHP and fuzzy TOPSIS to present the weights of decision makers with linguistic terms.

These methods mentioned above have made significant contributions to the determination of experts’ weights and aggregation of experts’ judgments in MAGDM. However, how to deal with the subjective and heuristic decisions of a group of experts in a simple and efficient way is still a question [34–38]. In order to address this question, an easy operation method in this paper is developed for determining weights of experts based on rough group decision.

Rough set theory, first proposed by Pawlak [11], is an effective and efficient tool to handle imprecision and vagueness information from DMs. As rough group decision originates from rough set theory, it can enable DMs to express true and objective evaluation without any priori information. Additionally, it can deal with a group of vague and subjective information at the same time.

The remainder of this paper is structured as follows. The following section gives a brief introduction to rough group decision. Then, we present the detailed description of the proposed method in group decision setting. Then, we compare the developed method in this study with other existing methods. Next, an illustrative example is given. Finally, the conclusions are made for the whole study.

## Determination of the rough group decision

Here, we shall introduce some concepts about the rough group decision.

**Definition 1** ([39]). Let *U* be a universe including all DMs’ decisions, *X* is an arbitrary decision of *U*. Assume that there is a set of each DM’s judgements on attributes over alternatives, $J=\left\{v_{ij}^{1},v_{ij}^{2},\ldots ,v_{ij}^{k},\ldots ,v_{ij}^{t}\right\}$, where *i* is the number of alternatives, *j* is the number of attributes and *t* is the number of DMs, *i* ∈ {1,2,…,*m*}, *j* ∈ {1,2,…,*n*}, *k* ∈ {1,2,…,*t*}, *t* > 0. Assume the elements of set *J* are in ascending order ($v_{ij}^{1}<v_{ij}^{2}<\ldots <v_{ij}^{k}<\ldots <v_{ij}^{t}$). Then, the lower approximation and the upper approximation of $v_{ij}^{k}$ are defined as:

$$\text{Lower approximation}:\;\underline{Apr}(v_{ij}^{k})=\cup \left\{X\in U|J(X)\leq v_{ij}^{k}\right\}$$ (1)

$$\text{Upper approximation}:\;\bar{Apr}(v_{ij}^{k})=\cup \left\{X\in U|J(X)\geq v_{ij}^{k}\right\}$$ (2)

In order to obtain the rough decision, the crisp decision $v_{ij}^{k}$, which contains vague and subjective information of a DM, should be converted into rough number form. As the geometric mean preserves the reciprocal property of pair-wise comparison matrixes, it is utilized to synthesize individual decisions from DMs.

**Definition 2** ([40]). A rough number is selected to represent the judgment $v_{ij}^{k}$, defined by its lower limit $\underline{Lim}(v_{ij}^{k})$ and upper limit $\bar{Lim}(v_{ij}^{k})$ as follows:

$$\underline{Lim}(v_{ij}^{k})={\left(\prod_{n=1}^{N_{L}}x\right)}^{1/N_{L}}$$ (3)

$$\bar{Lim}(v_{ij}^{k})={\left(\prod_{n=1}^{N_{U}}y\right)}^{1/N_{U}}$$ (4)

*x* and *y* are from the lower and upper approximation for $v_{ij}^{k}$. *N*_*L*_ and *N*_*U*_ are defined as the numbers of judgements from the lower approximation and upper approximation of $v_{ij}^{k}$.

**Definition 3** ([41]). The rough number form $RN(v_{ij}^{k})$ of $v_{ij}^{k}$ is obtained by using Eq (1)-Eq (4),

$$RN(v_{ij}^{k})=\left[\underline{Lim}(v_{ij}^{k}),\bar{Lim}(v_{ij}^{k})\right]=\left[v_{ij}^{kL},v_{ij}^{kU}\right]$$ (5)

where $v_{ij}^{kL}$ and $v_{ij}^{kU}$ are from the lower limit and upper limit of rough number $RN(v_{ij}^{k})$ in the *k*th decision matrix. The interval of boundary region (i.e. $v_{ij}^{kU}-v_{ij}^{kL}$) indicates the vagueness degree. That is, a smaller interval boundary to a rough number means more precise. Then, the crisp decision $v_{ij}^{k}$ is represented by the rough decision $RN(v_{ij}^{k})$.

**Definition 4**. In sum, the average rough interval $\bar{RN(J)}$ is obtained by using Eq (1)–Eq (5),

$$\bar{RN(J)}=\left[v_{ij}^{L},v_{ij}^{U}\right]$$ (6)

$$v_{ij}^{L}={\left(\prod_{k=1}^{t}v_{ij}^{kL}\right)}^{1/t}$$ (7)

$$v_{ij}^{U}={\left(\prod_{k=1}^{t}v_{ij}^{kU}\right)}^{1/t}$$ (8)

$v_{ij}^{L}$ and $v_{ij}^{U}$ are from the rough number $\left[v_{ij}^{L},\;v_{ij}^{U}\right]$. *t* is the number of experts. Then, a set of each DM’s decision, *J*, is represented by the average rough interval $\bar{RN(J)}$.

**Definition 5**. The average value of $\bar{RN(J)}$ is obtained as follows:

$${\left(\bar{RN(J)}\right)}^{*}=\frac{v_{ij}^{L}+v_{ij}^{U}}{2}$$ (9)

${\left(\bar{RN(J)}\right)}^{*}$, which is the median of the average rough interval $\bar{RN(J)}$, can reflect the common aspirations and consistent judgements of DMs with respect to the set *J*.

## Proposed approach to group decision making

In the following, the MAGDM problems under consideration with rough group decision shall be described in detail.

For convenience, assume *M* = {1,2,…,*m*}, *N* = {1,2,…,*n*} and *T* = {1,2,…,*t*} are three sets of indicators; *i* ∈ *M*, *j* ∈ *N*, *k* ∈ *T*. Assume there are m feasible alternatives *A*_*i*_ (*i* = 1,2,…,*m*) to be evaluated against n selection criteria *u*_*j*_ (*j* = 1,2,…,*n*) with n criteria’s weight *w*_*j*_ (*j* = 1,2,…,*n*), which satisfies 0 ≤ *w*_*j*_ ≤ 1 and $\sum_{j=1}^{n}w_{j}=1$. Assume *D* = {*d*_1_,*d*_2_,…,*d*_*t*_} is a finite set of DMs, *λ* = {*λ*_1_,*λ*_2_,…,*λ*_*t*_} is the weight vector of all DMs, which fulfils *λ*_*k*_ ≥ 0 and $\sum_{k=1}^{t}\lambda_{k}=1$.

### Standardization of the decision matrix

Invite DMs to give the relative importance of *m* feasible alternatives under *n* attributes by using the one-nine scale of AHP method. The decision matrix of the *k*th DM is as follows:

$$X_{k}={\left(x_{ij}^{k}\right)}_{m\times n}=\begin{array}{c} A_{1}\\ A_{2}\\ \vdots \\ A_{m} \end{array}\begin{array}{c} \begin{array}{cccc} u_{1} & \;u_{2} & \;\ldots & \;u_{n} \end{array}\\ \left[\begin{array}{cccc} x_{11}^{k} & x_{12}^{k} & \ldots & x_{1n}^{k}\\ x_{21}^{k} & x_{22}^{k} & \ldots & x_{2n}^{k}\\ \vdots & \vdots & \vdots & \vdots \\ x_{m1}^{k} & x_{m2}^{k} & \ldots & x_{mn}^{k} \end{array}\right] \end{array}$$ (10)

In general, MAGDM problems have benefit attributes (the larger the value is, the better) and cost attributes (the smaller the value is, the better). To acquire a dimensionless form, it is necessary to normalize each attribute value $x_{ij}^{k}$ in decision matrix *X*_*k*_ into a corresponding element $y_{ij}^{k}$ in normalized decision matrix *Y*_*k*_ by using Eqs (12) and (13) [34].

$$Y_{k}={\left(y_{ij}^{k}\right)}_{m\times n}=\begin{array}{c} A_{1}\\ A_{2}\\ \vdots \\ A_{m} \end{array}\begin{array}{c} \begin{array}{cccc} u_{1} & \;u_{2} & \;\ldots & \;u_{n} \end{array}\\ \left[\begin{array}{cccc} y_{11}^{k} & y_{12}^{k} & \ldots & y_{1n}^{k}\\ y_{21}^{k} & y_{22}^{k} & \ldots & y_{2n}^{k}\\ \vdots & \vdots & \vdots & \vdots \\ y_{m1}^{k} & y_{m2}^{k} & \ldots & y_{mn}^{k} \end{array}\right] \end{array}$$ (11)

where

$$y_{ij}^{k}=\frac{x_{ij}^{k}}{\sqrt{\sum_{i=1}^{m}{\left(x_{ij}^{k}\right)}^{2}}},\;\mathrm{for}\;\mathrm{benefit}\;\mathrm{attribute}\;x_{ij}^{k},\;i\in M,\;j\in N$$ (12)

and

$$y_{ij}^{k}=1-\frac{x_{ij}^{k}}{\sqrt{\sum_{i=1}^{m}{\left(x_{ij}^{k}\right)}^{2}}},\;\mathrm{for}\;\mathrm{cost}\;\mathrm{attribute}\;x_{ij}^{k},\;i\in M,\;j\in N$$ (13)

Then, it is clear that *u*_*j*_ ∈ [0,1], *j* ∈ *N*.

As the attributes’ weight vector $\left\{w_{1}^{k},w_{2}^{k},\ldots ,w_{n}^{k}\right\}$ is given by the *k*th DM, the weighted normalized decision matrix is constructed as

$$V_{k}={\left(v_{ij}^{k}\right)}_{m\times n}={\left(w_{j}^{k}y_{ij}^{k}\right)}_{m\times n}=\begin{array}{c} A_{1}\\ A_{2}\\ \vdots \\ A_{m} \end{array}\begin{array}{c} \begin{array}{cccc} u_{1} & \;u_{2} & \;\ldots & \;u_{n} \end{array}\\ \left[\begin{array}{cccc} v_{11}^{k} & v_{12}^{k} & \ldots & v_{1n}^{k}\\ v_{21}^{k} & v_{22}^{k} & \ldots & v_{2n}^{k}\\ \vdots & \vdots & \vdots & \vdots \\ v_{m1}^{k} & v_{m2}^{k} & \ldots & v_{mn}^{k} \end{array}\right] \end{array}$$ (14)

### Definition of DMs’ weights

Inspired by the idea of the rough group decision, the group decision matrix is built as follows:

$$\tilde{V}={\left({\tilde{v}}_{ij}\right)}_{m\times n}=\begin{array}{c} A_{1}\\ A_{2}\\ \vdots \\ A_{m} \end{array}\begin{array}{c} \begin{array}{cccc} u_{1} & \;u_{2} & \;\ldots & \;u_{n} \end{array}\\ \left[\begin{array}{cccc} {\tilde{v}}_{11} & {\tilde{v}}_{12} & \ldots & {\tilde{v}}_{1n}\\ {\tilde{v}}_{21} & {\tilde{v}}_{22} & \ldots & {\tilde{v}}_{2n}\\ \vdots & \vdots & \vdots & \vdots \\ {\tilde{v}}_{m1} & {\tilde{v}}_{m2} & \ldots & {\tilde{v}}_{mn} \end{array}\right] \end{array}$$ (15)

where ${\tilde{v}}_{ij}=\left\{v_{ij}^{1},v_{ij}^{2},\ldots ,v_{ij}^{k},\ldots ,v_{ij}^{t}\right\}$.

As mentioned above, ${\tilde{v}}_{ij}$ is a set of each DM’s judgements. Then, the average rough interval of ${\tilde{v}}_{ij}$ is obtained by applying Eq (1)-Eq (8). The rough group decision matrix *RV* is obtained as follows:

$$RV=\begin{array}{c} A_{1}\\ A_{2}\\ \vdots \\ A_{m} \end{array}\begin{array}{c} \begin{array}{cccc} u_{1} & \;u_{2} & \;\ldots & \;u_{n} \end{array}\\ \left[\begin{array}{cccc} \left[v_{11}^{L},v_{11}^{U}\right] & \left[v_{12}^{L},v_{12}^{U}\right] & \ldots & \left[v_{1n}^{L},v_{1n}^{U}\right]\\ \left[v_{21}^{L},v_{21}^{U}\right] & \left[v_{22}^{L},v_{22}^{U}\right] & \ldots & \left[v_{2n}^{L},v_{2n}^{U}\right]\\ \vdots & \vdots & \vdots & \vdots \\ \left[v_{m1}^{L},v_{m1}^{U}\right] & \left[v_{m2}^{L},v_{m2}^{U}\right] & \ldots & \left[v_{mn}^{L},v_{mn}^{U}\right] \end{array}\right] \end{array}$$ (16)

As known to all, TOPSIS has become a widely used technique for MAGDM. The main idea of TOPSIS is that the ideal alternative has the best level for all attributes, whereas the negative ideal has the worst level for all attributes. According to the idea of TOPSIS, we define RV^*^ as PIS for all individual decision matrixes with Definition 5 of previous section. Then, the average matrix of rough group decision matrix is obtained by using Eq (9).

$$RV^{+}=RV^{*}={\left(v_{ij}^{*}\right)}_{m\times n}=\begin{array}{c} A_{1}\\ A_{2}\\ \vdots \\ A_{m} \end{array}\begin{array}{c} \begin{array}{cccc} u_{1} & \;u_{2} & \;\ldots & \;u_{n} \end{array}\\ \left[\begin{array}{cccc} v_{11}^{*} & v_{12}^{*} & \ldots & v_{1n}^{*}\\ v_{21}^{*} & v_{22}^{*} & \ldots & v_{2n}^{*}\\ \vdots & \vdots & \vdots & \vdots \\ v_{m1}^{*} & v_{m2}^{*} & \ldots & v_{mn}^{*} \end{array}\right] \end{array}$$ (17)

where $v_{ij}^{*}=\left(v_{ij}^{L}+v_{ij}^{U}\right)/2$.

And then, from a TOPSIS method perspective, both the upper limit and the lower limit matrix of the rough group decision matrix are potential to have the farthest distance from the average matrix. Thus, we divided the NIS into two parts: L-NIS $RV_{L}^{-}$ and U-NIS $RV_{U}^{-}$.

$$RV_{L}^{-}={\left(v_{ij}^{L}\right)}_{m\times n}=\begin{array}{c} A_{1}\\ A_{2}\\ \vdots \\ A_{m} \end{array}\begin{array}{c} \begin{array}{cccc} u_{1} & \;u_{2} & \;\ldots & \;u_{n} \end{array}\\ \left[\begin{array}{cccc} v_{11}^{L} & v_{12}^{L} & \ldots & v_{1n}^{L}\\ v_{21}^{L} & v_{22}^{L} & \ldots & v_{2n}^{L}\\ \vdots & \vdots & \vdots & \vdots \\ v_{m1}^{L} & v_{m2}^{L} & \ldots & v_{mn}^{L} \end{array}\right] \end{array}$$ (18)

$$RV_{U}^{-}={\left(v_{ij}^{U}\right)}_{m\times n}=\begin{array}{c} A_{1}\\ A_{2}\\ \vdots \\ A_{m} \end{array}\begin{array}{c} \begin{array}{cccc} u_{1} & \;u_{2} & \;\ldots & \;u_{n} \end{array}\\ \left[\begin{array}{cccc} v_{11}^{U} & v_{12}^{U} & \ldots & v_{1n}^{U}\\ v_{21}^{U} & v_{22}^{U} & \ldots & v_{2n}^{U}\\ \vdots & \vdots & \vdots & \vdots \\ v_{m1}^{U} & v_{m2}^{U} & \ldots & v_{mn}^{U} \end{array}\right] \end{array}$$ (19)

where $v_{ij}^{L}$ and $v_{ij}^{U}$ are from the rough number $\left[v_{ij}^{L},v_{ij}^{U}\right]$.

The separation of each individual decision matrix *V*_*k*_ from the PIS RV^*^ is calculated as:

$$S_{k}^{+}=\left\|V_{k}-RV^{*}\right\|={\left(\sum_{i=1}^{m}\sum_{j=1}^{n}\left(v_{ij}^{k}-v_{ij}^{*}\right)\right)}^{\frac{1}{2}}$$ (20)

It is clear that the smaller the value of $S_{k}^{+}$ is, the more important the weight of the *k*th DM.

Similarly, the separation of each individual decision matrix *V*_*k*_ from the NISs $RV_{L}^{-}$ and $RV_{U}^{-}$ are calculated as:

$$S_{k}^{L-}=\left\|V_{k}-RV_{L}^{-}\right\|={\left(\sum_{i=1}^{m}\sum_{j=1}^{n}\left(v_{ij}^{k}-v_{ij}^{L}\right)\right)}^{\frac{1}{2}}$$ (21)

$$S_{k}^{U-}=\left\|V_{k}-RV_{U}^{-}\right\|={\left(\sum_{i=1}^{m}\sum_{j=1}^{n}\left(v_{ij}^{k}-v_{ij}^{U}\right)\right)}^{\frac{1}{2}}$$ (22)

It is clear that the larger the value of $S_{k}^{L-}$ and $S_{k}^{U-}$ are, the more important the weight of the *k*th DM.

Assuming that $S_{k}^{+}$, $S_{k}^{L-}$ and $S_{k}^{U-}$ are all under consideration, we define relative closeness to rank the weights of DMs. The relative closeness of the *k*th DM in relation to ideal solutions is defined as:

$$C_{k}=\frac{\max(S_{k}^{L-},S_{k}^{U-})}{S_{k}^{+}+\max(S_{k}^{L-},S_{k}^{U-})}$$ (23)

where $S_{k}^{+}\geq 0$, $S_{k}^{L-}\geq 0$ and $S_{k}^{U-}\geq 0$, so *C*_*k*_ ∈ [0,1]. Assume the decision matrix of the *k*th DM is the positive ideal solution; then, $S_{k}^{+}=0$ and *C*_*k*_ = 1. So if *C*_*k*_ = 1, the corresponding decision is absolutely the best decision.

According to Eqs (20)-(23), it can be inferred that if the individual matrix *V*_*k*_ is close to *RV*^+^, *V*_*k*_ is far from $RV_{L}^{-}$ and $RV_{U}^{-}$.

Therefore, we can define the weight of the *k*th DM as follows:

$$\lambda_{k}=\frac{C_{k}}{\sum_{k=1}^{t}C_{k}}$$ (24)

where *λ*_*k*_ ≥ 0 and $\sum_{k=1}^{t}\lambda_{k}=1$.

Then, we can rank the weights of DMs according to Eqs (23) and (24).

### Priority order of alternatives

With the weight of the *k*th DM, a group decision matrix *Y* is obtained by using the following formula

$$Y=\sum_{k=1}^{t}\lambda_{k}Y_{k}={\left(y_{ij}\right)}_{m\times n}$$ (25)

Then, use the aggregation formula

$$y_{i}=\sum_{j=1}^{n}y_{ij},\;i\in M$$ (26)

to summarize the *i*th row’s elements of *Y*. Then, the overall attribute value *y*_*i*_ of the alternative *A*_*i*_ is obtained.

According to the value *y*_*i*_, the priority order of those feasible alternatives can be ranked, and the best alternative can be chosen.

### The presented algorithm

As described above, a method for determining the DMs’ weights, based on the rough group decision, is shown as follows.

**Step 1.** Utilize Eq (12) and/or Eq (13) to normalize *X*_*k*_ into *Y*_*k*_ in Eq (11).
**Step 2.** Calculate the weighted normalized decision matrix *V*_*k*_ by multiplying $\left\{w_{1}^{k},w_{2}^{k},\ldots ,w_{n}^{k}\right\}$ and *Y*_*k*_ in Eq (14).
**Step 3.** Calculate the group decision matrix $\tilde{V}$ in Eq (15).
**Step 4.** Calculate the rough group decision matrix *RV* in Eq (16) by using Eq (1) to Eq (8).
**Step 5.** Determine the PIS and NISs of all individual decisions, *RV*^+^, $RV_{L}^{-}$ and $RV_{U}^{-}$, by using Eq (17)-Eq (19).
**Step 6.** Calculate the separation from each individual decision to the ideal decisions, $S_{k}^{+}$, $S_{k}^{L-}$ and $S_{k}^{U-}$, by applying Eq (20)-Eq (22).
**Step 7.** Calculate the relative closeness to the ideal solutions by using Eq (23).
**Step 8.** Calculate the DMs’ weight vector *λ* = (*λ*_1_,*λ*_2_,…*λ*_*t*_)^*T*^ by using Eq (24).
**Step 9.** Calculate the overall decision matrix by using Eq (25), based on the DMs’ weight vector *λ* = (*λ*_1_,*λ*_2_,…*λ*_*t*_)^*T*^.
**Step 10.** Summarize each line’s elements of the collective decision matrix in Eq (26) and obtain an overall assessment value for each alternative.
**Step 11.** Rank the preference order of all alternatives according to their total assessment values.

**Fig 1:**
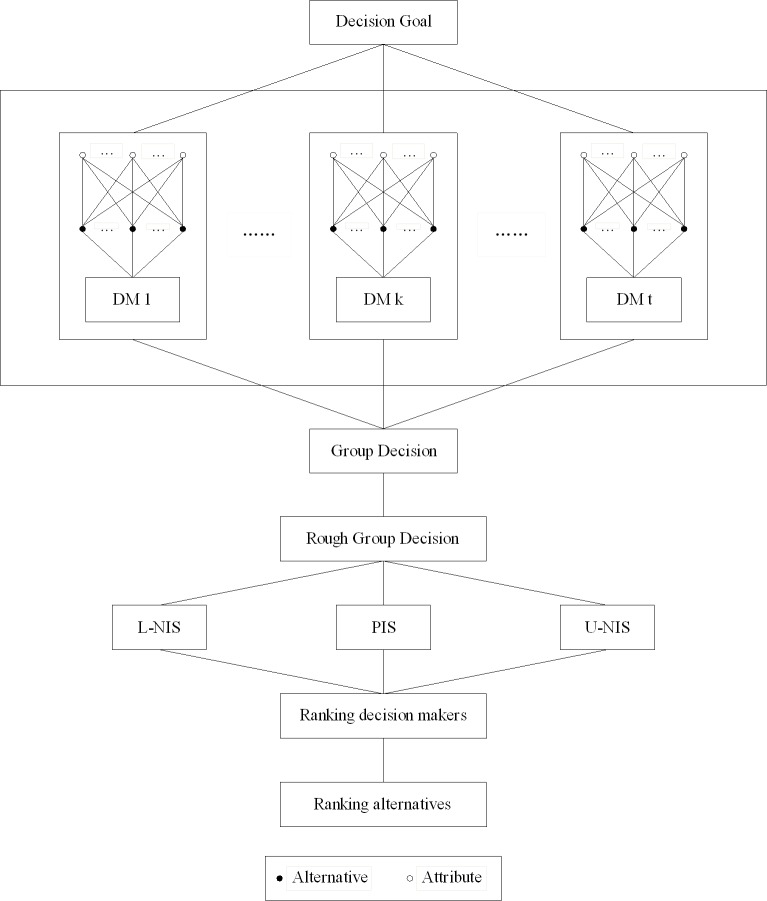
Hierarchical structure of the proposed approach.

The hierarchical structure of the proposed approach is summarized in Fig 1.

## Comparisons between the proposed approach and existing approaches

In the following, we compared three approaches, the proposed approach, the method of Ye and Li [42] and the extended TOPSIS method of Yue [31].

Table 1 presents the differences between the two methods, the proposed method and the extended TOPSIS method of Ye and Li. First, the PIS and NIS are derived from alternatives, which are vectors, while in this paper, they are derived from rough group decision matrix, which are matrixes. Second, each DM’s weight is different and determined by the distances from his/her decision matrix to PIS and NISs in this paper. That is to say, the weight of each DM is defined by the given data, not pre-defined, and reflects the gap between his/her preference and group preference to the feasible alternatives on attributes objectively. In addition, the developed approach’s procedure in Fig 1 is simple and clear for high-dimensional data analysis in group setting.

**Table 1:** Comparison with the extended TOPSIS of Ye and Li.

Characteristics	Method of Ye and Li	Rough set group approach
Evaluation objective	Ranking of a group of alternatives	Ranking of a group of DMs
No. of DMs	More than one	More than one
Weights on attributes	Given	Given
PIS	The best alternative represented by a vector	The best decision represented by the average matrix of rough group decision
NIS	The worst alternative represented by a vector	The worst decision represented by the upper limit and lower limit matrix of rough group decision
Core process	The separation from each alternative to PIS and NIS	The separation from each individual decision to PIS and NISs
Weights on DMs	Same	Different

In the method of Yue, the three benchmark matrixes (PIS, L-NIS and U-NIS) are defined through aggregation of DMs’ decision information by using TOPSIS, while in this paper these matrixes are defined by rough group decision, which are based on rough number and rough boundary interval. The average rough boundary interval in Eq (17) from rough boundary intervals can reflect the vagueness degree of all DMs to attributes of alternatives. From this point of view, the smaller the interval, the lower the vagueness degree. In addition, both of the two methods take a group effect with PIS and NISs into account. That is, if the decision matrix is far away from the NISs and close to the PIS, the decision is better. Therefore, the better the decision is, the more the DM’s weight. The comparisons mentioned above are shown in Table 2.

**Table 2:** Comparison with the extended TOPSIS of Yue.

Characteristics	Method of Yue	Rough set group approach
Evaluation objective	Ranking of a group of DMs	Ranking of a group of DMs
No. of DMs	More than one	More than one
Mathematical principle	Arithmetic average theory	Rough set theory
PIS	The best decision represented by the average value of group decision	The best decision represented by the average matrix of rough group decision
NIS	The worst decision represented by the max value and min value of group decision	The worst decision represented by the upper limit and lower limit matrix of rough group decision
relative closeness	$C_{k}=\frac{S_{k}^{l-}+S_{k}^{r-}}{S_{k}^{+}+S_{k}^{l-}+S_{k}^{r-}}$	$C_{k}=\frac{\max(S_{k}^{L-},S_{k}^{U-})}{S_{k}^{+}+\max(S_{k}^{L-},S_{k}^{U-})}$
Goal	Priority order of alternatives	Priority order of alternatives

## Illustrative example

In the following, the proposed method shall be applied to a human resources management [43]. A company wants to hire an on-line business manager. Therefore, the company proposes several relevant tests, which are regarded as the evaluated benefit criterias. These tests include knowledge tests and skill tests. In this manager selection, there are 17 available candidates (marked by A_1_,A_2,_…,A_17_). Then, there are four experts (marked by d_1_,d_2_,d_3_,d_4_) for the manager selection to carry out knowledge tests and skill tests. The original data of panel interview and 1-on-1 interview tests from four experts are list in Table 3.

**Table 3:** Decision matrixes of example-subjective attributes.

No. of candidates	*X*_*1*_		*X*_*2*_		*X*_*3*_		*X*_*4*_	
	Panel interview	1-on-1 interview	Panel interview	1-on-1 interview	Panel interview	1-on-1 interview	Panel interview	1-on-1 interview
1	80	75	85	80	75	70	90	85
2	65	75	60	70	70	77	60	70
3	90	85	80	85	80	90	90	95
4	65	70	55	60	68	72	62	72
5	75	80	75	80	50	55	70	75
6	80	80	75	85	77	82	75	75
7	65	70	70	60	65	72	67	75
8	70	60	75	65	75	67	82	85
9	80	85	95	85	90	85	90	92
10	70	75	75	80	68	78	65	70
11	50	60	62	65	60	65	65	70
12	60	65	65	75	50	60	45	50
13	75	75	80	80	65	75	70	75
14	80	70	75	72	80	70	75	75
15	70	65	75	70	65	70	60	65
16	90	95	92	90	85	80	88	90
17	80	85	70	75	75	80	70	75

In accordance with the suggested steps mentioned above, each decision matrix given by experts in Table 3 shall be normalized to achieve nondimensionalization. Because of the benefit attributes of Table 3, we first normalize Table 3 into four normalized decision matrixes of Table 4 according to Step 1. In the normalized decision matrixes of Table 4, *X*_1_, *X*_2_, *X*_3_, *X*_4_ shall be marked by *Y*_1_, *Y*_2_, *Y*_3_, *Y*_4_, respectively.

**Table 4:** Normalized decision matrixes.

No.	*Y*_*1*_		*Y*_*2*_		*Y*_*3*_		*Y*_*4*_	
	Panel interview	1-on-1 interview	Panel interview	1-on-1 interview	Panel interview	1-on-1 interview	Panel interview	1-on-1 interview
1	0.2624	0.2416	0.2747	0.2565	0.2552	0.2297	0.2988	0.2683
2	0.2132	0.2416	0.1939	0.2245	0.2382	0.2526	0.1992	0.2209
3	0.2952	0.2738	0.2585	0.2726	0.2722	0.2953	0.2988	0.2998
4	0.2132	0.2255	0.1777	0.1924	0.2314	0.2362	0.2058	0.2272
5	0.2460	0.2577	0.2424	0.2565	0.1702	0.1805	0.2324	0.2367
6	0.2624	0.2577	0.2424	0.2726	0.2620	0.2690	0.2490	0.2367
7	0.2132	0.2255	0.2262	0.1924	0.2212	0.2362	0.2224	0.2367
8	0.2296	0.1933	0.2424	0.2084	0.2552	0.2198	0.2722	0.2683
9	0.2624	0.2738	0.3070	0.2726	0.3063	0.2789	0.2988	0.2904
10	0.2296	0.2416	0.2424	0.2565	0.2314	0.2559	0.2158	0.2209
11	0.1640	0.1933	0.2004	0.2084	0.2042	0.2133	0.2158	0.2209
12	0.1968	0.2094	0.2101	0.2405	0.1702	0.1969	0.1494	0.1578
13	0.2460	0.2416	0.2585	0.2565	0.2212	0.2461	0.2324	0.2367
14	0.2624	0.2255	0.2424	0.2309	0.2722	0.2297	0.2490	0.2367
15	0.2296	0.2094	0.2424	0.2245	0.2212	0.2297	0.1992	0.2051
16	0.2952	0.3061	0.2973	0.2886	0.2893	0.2625	0.2922	0.2840
17	0.2624	0.2738	0.2262	0.2405	0.2552	0.2625	0.2324	0.2367

Then, the weights of attributes are shown in Table 5, which are given by the four experts.

**Table 5:** Weights on attributes of example.

No.	Attributes	The weights of the group			
		*d*_1_	*d*_2_	*d*_3_	*d*_4_
1	Panel interview	0.5243	0.4574	0.4160	0.4503
2	1-on-1 interview	0.4757	0.5426	0.5840	0.5497

By using Step 2, each column vector of the normalized decision matrix is multiplied by the associated attributes’ weight vector given by each expert in Table 5. Therefore, the weighted normalized decision matrixes are obtained in Table 6.

**Table 6:** Weights normalized decision matrixes.

No.	*Y*_*1*_		*Y*_*2*_		*Y*_*3*_		*Y*_*4*_	
	Panel interview	1-on-1 interview	Panel interview	1-on-1 interview	Panel interview	1-on-1 interview	Panel interview	1-on-1 interview
1	0.1376	0.1149	0.1256	0.1392	0.1062	0.1341	0.1345	0.1475
2	0.1118	0.1149	0.0887	0.1218	0.0991	0.1475	0.0897	0.1214
3	0.1548	0.1303	0.1182	0.1479	0.1133	0.1724	0.1345	0.1648
4	0.1118	0.1073	0.0813	0.1044	0.0963	0.1380	0.0927	0.1249
5	0.1290	0.1226	0.1109	0.1392	0.0708	0.1054	0.1046	0.1301
6	0.1376	0.1226	0.1109	0.1479	0.1090	0.1571	0.1121	0.1301
7	0.1118	0.1073	0.1035	0.1044	0.0920	0.1380	0.1002	0.1301
8	0.1204	0.0920	0.1109	0.1131	0.1062	0.1284	0.1226	0.1475
9	0.1376	0.1303	0.1404	0.1479	0.1274	0.1629	0.1345	0.1596
10	0.1204	0.1149	0.1109	0.1392	0.0963	0.1495	0.0972	0.1214
11	0.0860	0.0920	0.0916	0.1131	0.0849	0.1245	0.0972	0.1214
12	0.1032	0.0996	0.0961	0.1305	0.0708	0.1150	0.0673	0.0867
13	0.1290	0.1149	0.1182	0.1392	0.0920	0.1437	0.1046	0.1301
14	0.1376	0.1073	0.1109	0.1253	0.1133	0.1341	0.1121	0.1301
15	0.1204	0.0996	0.1109	0.1218	0.0920	0.1341	0.0897	0.1128
16	0.1548	0.1456	0.1360	0.1566	0.1203	0.1533	0.1316	0.1561
17	0.1376	0.1303	0.1035	0.1305	0.1062	0.1533	0.1046	0.1301

By using Step 3 and Step 4, we can calculate the rough group decision matrix from the weighted normalized decision matrixes. Next, these important matrixes (*RV*^+^, $RV_{L}^{-}$ and $RV_{U}^{-}$) are shown in Table 7 by using Step 5.

**Table 7:** Ideal solutions.

No.	PIS *RV*^+^		L-NIS $RV_{L}^{-}$		U-NIS $RV_{U}^{-}$	
	Panel interview	1-on-1 interview	Panel interview	1-on-1 interview	Panel interview	1-on-1 interview
1	0.1249	0.1331	0.1169	0.1252	0.1328	0.1410
2	0.0975	0.1268	0.0917	0.1195	0.1033	0.1340
3	0.1302	0.1528	0.1198	0.1420	0.1405	0.1637
4	0.0952	0.1185	0.0881	0.1099	0.1024	0.1272
5	0.1014	0.1235	0.0872	0.1152	0.1157	0.1319
6	0.1179	0.1391	0.1116	0.1300	0.1243	0.1481
7	0.1017	0.1196	0.0970	0.1105	0.1064	0.1286
8	0.1148	0.1189	0.1104	0.1052	0.1191	0.1327
9	0.1348	0.1492	0.1316	0.1408	0.1379	0.1575
10	0.1062	0.1309	0.0999	0.1220	0.1125	0.1399
11	0.0900	0.1114	0.0869	0.1032	0.0932	0.1197
12	0.0835	0.1072	0.0738	0.0963	0.0933	0.1182
13	0.1103	0.1312	0.1009	0.1239	0.1197	0.1385
14	0.1190	0.1233	0.1131	0.1167	0.1250	0.1299
15	0.1030	0.1166	0.0949	0.1081	0.1111	0.1251
16	0.1356	0.1526	0.1275	0.1498	0.1437	0.1553
17	0.1136	0.1367	0.1061	0.1316	0.1211	0.1418

By using Step 6, the distances from each weighted normalized decision matrix to the ideal solutions (*RV*^+^, $RV_{L}^{-}$ and $RV_{U}^{-}$) are calculated. The results are summarized in Table 8.

Next, the relative closeness by using Step 7, the weight vector of experts by using Step 8, and experts’ priority ranking are calculated, respectively. These results mentioned above are all list in Table 8. The final experts’ priority ranking obtained by the rough group decision method is shown as

$$d_{2}>d_{4}>d_{3}>d_{1}.$$

**Table 8:** Separations, relative closeness, weights and ranking of experts.

DMs	$S_{k}^{+}$	$S_{k}^{L-}$	$S_{k}^{U-}$	*C*_*k*_	*λ*_*k*_	Ranking
*d*_1_	0.0964	0.1076	0.1074	0.5276	0.2370	4
*d*_2_	0.0521	0.0742	0.0669	0.5874	0.2639	1
*d*_3_	0.0827	0.0969	0.0940	0.5395	0.2424	3
*d*_4_	0.0578	0.0771	0.0728	0.5714	0.2567	2

The 6th column of Table 8 shows the weights of four invited experts. By Step 10, the Eq (25) is used to combine each DM’s decision to the collective decisions, which are shown in the column 2 and 3 of Table 9. Next, the overall evaluations of 17 candidates are shown in column 4 by summarizing all data in each line of columns 2 and 3 of Table 9. Finally, the ranking for these candidates are obtained in the last column of Table 9. It is clear that the 16th candidate ranks the first, and the 12th candidate ranks the last.

**Table 9:** Integrated assessment of 17 candidates.

No. of candidates	Panel interview	1-on-1 interview	Sum	Ranking
1	0.1260	0.1343	0.2604	4
2	0.0969	0.1263	0.2233	12
3	0.1299	0.1540	0.2839	3
4	0.0951	0.1185	0.2136	15
5	0.1039	0.1247	0.2286	11
6	0.1171	0.1396	0.2566	5
7	0.1018	0.1198	0.2216	13
8	0.1150	0.1206	0.2356	10
9	0.1351	0.1503	0.2854	2
10	0.1061	0.1314	0.2374	9
11	0.0901	0.1130	0.2031	16
12(#)	0.0842	0.1082	0.1924	17
13	0.1109	0.1322	0.2432	7
14	0.1181	0.1244	0.2425	8
15	0.1031	0.1172	0.2203	14
16(*)	0.1355	0.1531	0.2886	1
17	0.1125	0.1359	0.2484	6

Note: “*” and “#” mark the first and the last candidate, respectively.

## Conclusions

This paper designs a novel method to determine the weights of experts based on rough group decision. The proposed approach utilizes rough group decision to aggregate the subjective and heuristic information of experts. The validation of this method in a human resources selection indicates that it can be regarded as an objective and effective evaluation tool in group decision-making.

By contrast, the rough group method can effectively manage the subjectivity of experts in decision process and reflect the vagueness of experts objectively. Due to the amount of information, it will be easier and faster to solve these problems with software MATLAB. Although the method in this paper provides a simple and effective mechanism for weights of experts in group decision setting, it is only useful for real number form of attributes. Therefore, we shall extend the proposed approach to support other forms information on attributes, such as linguistic variables or fuzzy numbers in future work.

## Supporting information

S1 File **This file contains all Supporting Figures A and Tables A-I. Figure A in S1 File**. Figure A shows the hierarchical structure of the proposed approach. **Table A in S1 File**. Table A presents the differences and similarities between the extended TOPSIS of Ye and Li and the proposed method. **Table B in S1 File**. Table B presents the differences and similarities between the extended TOPSIS of Yue and the proposed method. **Table C in S1 File**. Table C lists the original data from four experts. **Table D in S1 File**. Table D shows the normalized decision matrixes. **Table E in S1 File**. Table E presents the weights of attributes given by the four experts. **Table F in S1 File**. Table F lists the weights normalized decision matrixes. **Table G in S1 File**. Table G presents the ideal solutions for all individual decision matrixes. **Table H in S1 File**. Table H shows the separations, relative closeness, weights and ranking of four experts. **Table I in S1 File**. Table I lists the integrated assessment of 17 candidates.
(DOCX)
