# Supplementary material for: A rough set approach for determining weights of decision makers in group decision making
Source: PLoS One. 2017 Feb 24;12(2):e0172679. doi: 10.1371/journal.pone.0172679 (PMC5325315; doi:10.1371/journal.pone.0172679)
Supplement: S1 File — This file contains all Supporting Figures A and Tables A-I. Figure A in S1 File. Figure A shows the hierarchical structure of the proposed approach. Table A in S1 File. Table A presents the differences and similarities between the extended TOPSIS of Ye and Li and the proposed method. Table B in S1 File. Table B presents the differences and similarities between the extended TOPSIS of Yue and the proposed method. Table C in S1 File. Table C lists the original data from four experts. Table D in S1 File. Table D shows the normalized decision matrixes. Table E in S1 File. Table E presents the weights of attributes given by the four experts. Table F in S1 File. Table F lists the weights normalized decision matrixes. Table G in S1 File. Table G presents the ideal solutions for all individual decision matrixes. Table H in S1 File. Table H shows the separations, relative closeness, weights and ranking of four experts. Table I in S1 File. Table I lists the integrated assessment of 17 candidates. (DOCX) [file pone.0172679.s001.docx]

**Fig A. Hierarchical structure of the proposed approach.**

**Table A. Comparison with the extended TOPSIS of Ye and Li.**

| Characteristics | Method of Ye and Li | Rough set group approach |
| --- | --- | --- |
| Evaluation objective | Ranking of a group of alternatives | Ranking of a group of DMs |
| No. of DMs | More than one | More than one |
| Weights on attributes | Given | Given |
| PIS | The best alternative represented by a vector | The best decision represented by the average matrix of rough group decision |
| NIS | The worst alternative represented by a vector | The worst decision represented by the upper limit and lower limit matrix of rough group decision |
| Core process | The separation from each alternative to PIS and NIS | The separation from each individual decision to PIS and NISs |
| Weights on DMs | Same | Different |

**Table B. Comparison with the extended TOPSIS of Yue.**

| Characteristics | Method of Yue | Rough set group approach |
| --- | --- | --- |
| Evaluation objective | Ranking of a group of DMs | Ranking of a group of DMs |
| No. of DMs | More than one | More than one |
| Mathematical  principle | Arithmetic average theory | Rough set theory |
| PIS | The best decision represented by the average value of group decision | The best decision represented by the average matrix of rough group decision |
| NIS | The worst decision represented by the max value and min value of group decision | The worst decision represented by the upper limit and lower limit matrix of rough group decision |
| relative closeness | $C_{k}=\frac{S_{k}^{l-}+S_{k}^{r-}}{S_{k}^{+}+S_{k}^{l-}+S_{k}^{r-}}$ | $C_{k}=\frac{\max(S_{k}^{L-},S_{k}^{U-})}{S_{k}^{+}+\max(S_{k}^{L-},S_{k}^{U-})}$ |
| Goal | Priority order of alternatives | Priority order of alternatives |

**Table C. Decision matrixes of example-subjective attributes.**

| No. of  candidates | *X_1_* | | *X_2_* | | *X_3_* | | *X_4_* | |
| --- | --- | --- | --- | --- | --- | --- | --- | --- |
|  | Panel  interview | 1-on-1  interview | Panel  interview | 1-on-1  interview | Panel  interview | 1-on-1  interview | Panel  interview | 1-on-1  interview |
| 1 | 80 | 75 | 85 | 80 | 75 | 70 | 90 | 85 |
| 2 | 65 | 75 | 60 | 70 | 70 | 77 | 60 | 70 |
| 3 | 90 | 85 | 80 | 85 | 80 | 90 | 90 | 95 |
| 4 | 65 | 70 | 55 | 60 | 68 | 72 | 62 | 72 |
| 5 | 75 | 80 | 75 | 80 | 50 | 55 | 70 | 75 |
| 6 | 80 | 80 | 75 | 85 | 77 | 82 | 75 | 75 |
| 7 | 65 | 70 | 70 | 60 | 65 | 72 | 67 | 75 |
| 8 | 70 | 60 | 75 | 65 | 75 | 67 | 82 | 85 |
| 9 | 80 | 85 | 95 | 85 | 90 | 85 | 90 | 92 |
| 10 | 70 | 75 | 75 | 80 | 68 | 78 | 65 | 70 |
| 11 | 50 | 60 | 62 | 65 | 60 | 65 | 65 | 70 |
| 12 | 60 | 65 | 65 | 75 | 50 | 60 | 45 | 50 |
| 13 | 75 | 75 | 80 | 80 | 65 | 75 | 70 | 75 |
| 14 | 80 | 70 | 75 | 72 | 80 | 70 | 75 | 75 |
| 15 | 70 | 65 | 75 | 70 | 65 | 70 | 60 | 65 |
| 16 | 90 | 95 | 92 | 90 | 85 | 80 | 88 | 90 |
| 17 | 80 | 85 | 70 | 75 | 75 | 80 | 70 | 75 |

**Table D. Normalized decision matrixes.**

| No. | *Y_1_* | | *Y_2_* | | *Y_3_* | | *Y_4_* | |
| --- | --- | --- | --- | --- | --- | --- | --- | --- |
|  | Panel  interview | 1-on-1  interview | Panel  interview | 1-on-1  interview | Panel  interview | 1-on-1  interview | Panel  interview | 1-on-1  interview |
| 1 | 0.2624 | 0.2416 | 0.2747 | 0.2565 | 0.2552 | 0.2297 | 0.2988 | 0.2683 |
| 2 | 0.2132 | 0.2416 | 0.1939 | 0.2245 | 0.2382 | 0.2526 | 0.1992 | 0.2209 |
| 3 | 0.2952 | 0.2738 | 0.2585 | 0.2726 | 0.2722 | 0.2953 | 0.2988 | 0.2998 |
| 4 | 0.2132 | 0.2255 | 0.1777 | 0.1924 | 0.2314 | 0.2362 | 0.2058 | 0.2272 |
| 5 | 0.2460 | 0.2577 | 0.2424 | 0.2565 | 0.1702 | 0.1805 | 0.2324 | 0.2367 |
| 6 | 0.2624 | 0.2577 | 0.2424 | 0.2726 | 0.2620 | 0.2690 | 0.2490 | 0.2367 |
| 7 | 0.2132 | 0.2255 | 0.2262 | 0.1924 | 0.2212 | 0.2362 | 0.2224 | 0.2367 |
| 8 | 0.2296 | 0.1933 | 0.2424 | 0.2084 | 0.2552 | 0.2198 | 0.2722 | 0.2683 |
| 9 | 0.2624 | 0.2738 | 0.3070 | 0.2726 | 0.3063 | 0.2789 | 0.2988 | 0.2904 |
| 10 | 0.2296 | 0.2416 | 0.2424 | 0.2565 | 0.2314 | 0.2559 | 0.2158 | 0.2209 |
| 11 | 0.1640 | 0.1933 | 0.2004 | 0.2084 | 0.2042 | 0.2133 | 0.2158 | 0.2209 |
| 12 | 0.1968 | 0.2094 | 0.2101 | 0.2405 | 0.1702 | 0.1969 | 0.1494 | 0.1578 |
| 13 | 0.2460 | 0.2416 | 0.2585 | 0.2565 | 0.2212 | 0.2461 | 0.2324 | 0.2367 |
| 14 | 0.2624 | 0.2255 | 0.2424 | 0.2309 | 0.2722 | 0.2297 | 0.2490 | 0.2367 |
| 15 | 0.2296 | 0.2094 | 0.2424 | 0.2245 | 0.2212 | 0.2297 | 0.1992 | 0.2051 |
| 16 | 0.2952 | 0.3061 | 0.2973 | 0.2886 | 0.2893 | 0.2625 | 0.2922 | 0.2840 |
| 17 | 0.2624 | 0.2738 | 0.2262 | 0.2405 | 0.2552 | 0.2625 | 0.2324 | 0.2367 |

**Table E. Weights on attributes of example.**

| No. | Attributes | The weights of the group | | | |
| --- | --- | --- | --- | --- | --- |
|  |  | *d*_1_ | *d*_2_ | *d*_3_ | *d*_4_ |
| 1 | Panel interview | 0.5243 | 0.4574 | 0.4160 | 0.4503 |
| 2 | 1-on-1 interview | 0.4757 | 0.5426 | 0.5840 | 0.5497 |

**Table F. Weights normalized decision matrixes.**

| No. | *Y_1_* | | *Y_2_* | | *Y_3_* | | *Y_4_* | |
| --- | --- | --- | --- | --- | --- | --- | --- | --- |
|  | Panel  interview | 1-on-1  interview | Panel  interview | 1-on-1  interview | Panel  interview | 1-on-1  interview | Panel  interview | 1-on-1  interview |
| 1 | 0.1376 | 0.1149 | 0.1256 | 0.1392 | 0.1062 | 0.1341 | 0.1345 | 0.1475 |
| 2 | 0.1118 | 0.1149 | 0.0887 | 0.1218 | 0.0991 | 0.1475 | 0.0897 | 0.1214 |
| 3 | 0.1548 | 0.1303 | 0.1182 | 0.1479 | 0.1133 | 0.1724 | 0.1345 | 0.1648 |
| 4 | 0.1118 | 0.1073 | 0.0813 | 0.1044 | 0.0963 | 0.1380 | 0.0927 | 0.1249 |
| 5 | 0.1290 | 0.1226 | 0.1109 | 0.1392 | 0.0708 | 0.1054 | 0.1046 | 0.1301 |
| 6 | 0.1376 | 0.1226 | 0.1109 | 0.1479 | 0.1090 | 0.1571 | 0.1121 | 0.1301 |
| 7 | 0.1118 | 0.1073 | 0.1035 | 0.1044 | 0.0920 | 0.1380 | 0.1002 | 0.1301 |
| 8 | 0.1204 | 0.0920 | 0.1109 | 0.1131 | 0.1062 | 0.1284 | 0.1226 | 0.1475 |
| 9 | 0.1376 | 0.1303 | 0.1404 | 0.1479 | 0.1274 | 0.1629 | 0.1345 | 0.1596 |
| 10 | 0.1204 | 0.1149 | 0.1109 | 0.1392 | 0.0963 | 0.1495 | 0.0972 | 0.1214 |
| 11 | 0.0860 | 0.0920 | 0.0916 | 0.1131 | 0.0849 | 0.1245 | 0.0972 | 0.1214 |
| 12 | 0.1032 | 0.0996 | 0.0961 | 0.1305 | 0.0708 | 0.1150 | 0.0673 | 0.0867 |
| 13 | 0.1290 | 0.1149 | 0.1182 | 0.1392 | 0.0920 | 0.1437 | 0.1046 | 0.1301 |
| 14 | 0.1376 | 0.1073 | 0.1109 | 0.1253 | 0.1133 | 0.1341 | 0.1121 | 0.1301 |
| 15 | 0.1204 | 0.0996 | 0.1109 | 0.1218 | 0.0920 | 0.1341 | 0.0897 | 0.1128 |
| 16 | 0.1548 | 0.1456 | 0.1360 | 0.1566 | 0.1203 | 0.1533 | 0.1316 | 0.1561 |
| 17 | 0.1376 | 0.1303 | 0.1035 | 0.1305 | 0.1062 | 0.1533 | 0.1046 | 0.1301 |

**Table G. Ideal solutions.**

| No. | PIS $RV^{+}$ | | L-NIS $RV_{L}^{-}$ | | U-NIS $RV_{U}^{-}$ | |
| --- | --- | --- | --- | --- | --- | --- |
|  | Panel  interview | 1-on-1  interview | Panel  interview | 1-on-1  interview | Panel  interview | 1-on-1  interview |
| 1 | 0.1249 | 0.1331 | 0.1169 | 0.1252 | 0.1328 | 0.1410 |
| 2 | 0.0975 | 0.1268 | 0.0917 | 0.1195 | 0.1033 | 0.1340 |
| 3 | 0.1302 | 0.1528 | 0.1198 | 0.1420 | 0.1405 | 0.1637 |
| 4 | 0.0952 | 0.1185 | 0.0881 | 0.1099 | 0.1024 | 0.1272 |
| 5 | 0.1014 | 0.1235 | 0.0872 | 0.1152 | 0.1157 | 0.1319 |
| 6 | 0.1179 | 0.1391 | 0.1116 | 0.1300 | 0.1243 | 0.1481 |
| 7 | 0.1017 | 0.1196 | 0.0970 | 0.1105 | 0.1064 | 0.1286 |
| 8 | 0.1148 | 0.1189 | 0.1104 | 0.1052 | 0.1191 | 0.1327 |
| 9 | 0.1348 | 0.1492 | 0.1316 | 0.1408 | 0.1379 | 0.1575 |
| 10 | 0.1062 | 0.1309 | 0.0999 | 0.1220 | 0.1125 | 0.1399 |
| 11 | 0.0900 | 0.1114 | 0.0869 | 0.1032 | 0.0932 | 0.1197 |
| 12 | 0.0835 | 0.1072 | 0.0738 | 0.0963 | 0.0933 | 0.1182 |
| 13 | 0.1103 | 0.1312 | 0.1009 | 0.1239 | 0.1197 | 0.1385 |
| 14 | 0.1190 | 0.1233 | 0.1131 | 0.1167 | 0.1250 | 0.1299 |
| 15 | 0.1030 | 0.1166 | 0.0949 | 0.1081 | 0.1111 | 0.1251 |
| 16 | 0.1356 | 0.1526 | 0.1275 | 0.1498 | 0.1437 | 0.1553 |
| 17 | 0.1136 | 0.1367 | 0.1061 | 0.1316 | 0.1211 | 0.1418 |

**Table H. Separations, relative closeness, weights and ranking of experts.**

| DMs | $S_{k}^{+}$ | $S_{k}^{L-}$ | $S_{k}^{U-}$ | $C_{k}$ | $\lambda_{k}$ | Ranking |
| --- | --- | --- | --- | --- | --- | --- |
| *d*_1_ | 0.0964 | 0.1076 | 0.1074 | 0.5276 | 0.2370 | 4 |
| *d*_2_ | 0.0521 | 0.0742 | 0.0669 | 0.5874 | 0.2639 | 1 |
| *d*_3_ | 0.0827 | 0.0969 | 0.0940 | 0.5395 | 0.2424 | 3 |
| *d*_4_ | 0.0578 | 0.0771 | 0.0728 | 0.5714 | 0.2567 | 2 |

**Table I. Integrated assessment of 17 candidates.**

| No. of candidates | Panel interview | 1-on-1 interview | Sum | Ranking |
| --- | --- | --- | --- | --- |
| 1 | 0.1260 | 0.1343 | 0.2604 | 4 |
| 2 | 0.0969 | 0.1263 | 0.2233 | 12 |
| 3 | 0.1299 | 0.1540 | 0.2839 | 3 |
| 4 | 0.0951 | 0.1185 | 0.2136 | 15 |
| 5 | 0.1039 | 0.1247 | 0.2286 | 11 |
| 6 | 0.1171 | 0.1396 | 0.2566 | 5 |
| 7 | 0.1018 | 0.1198 | 0.2216 | 13 |
| 8 | 0.1150 | 0.1206 | 0.2356 | 10 |
| 9 | 0.1351 | 0.1503 | 0.2854 | 2 |
| 10 | 0.1061 | 0.1314 | 0.2374 | 9 |
| 11 | 0.0901 | 0.1130 | 0.2031 | 16 |
| 12(#) | 0.0842 | 0.1082 | 0.1924 | 17 |
| 13 | 0.1109 | 0.1322 | 0.2432 | 7 |
| 14 | 0.1181 | 0.1244 | 0.2425 | 8 |
| 15 | 0.1031 | 0.1172 | 0.2203 | 14 |
| 16(*) | 0.1355 | 0.1531 | 0.2886 | 1 |
| 17 | 0.1125 | 0.1359 | 0.2484 | 6 |

Note: “*” and “#” mark the first and the last candidate, respectively.
